# Genotype at the P554L Variant of the Hexose-6 Phosphate Dehydrogenase Gene Is Associated with Carotid Intima-Medial Thickness

**DOI:** 10.1371/journal.pone.0023248

**Published:** 2011-08-12

**Authors:** Thahira J. Rahman, Elizabeth A. Walker, Bongani M. Mayosi, Darroch H. Hall, Peter J. Avery, John M. C. Connell, Hugh Watkins, Paul M. Stewart, Bernard Keavney

**Affiliations:** 1 Institute of Genetic Medicine, Newcastle University, Newcastle upon Tyne, United Kingdom; 2 Department of Medicine, University of Birmingham, Birmingham, United Kingdom; 3 Department of Medicine, University of Cape Town, Cape Town, South Africa; 4 School of Mathematics and Statistics, Newcastle University, Newcastle upon Tyne, United Kingdom; 5 Department of Medicine, University of Dundee, Dundee, United Kingdom; 6 Department of Cardiovascular Medicine, Oxford University, Oxford, United Kingdom; Biomedical Research Institute, United States of America

## Abstract

**Objective:**

The combined thickness of the intima and media of the carotid artery (carotid intima-medial thickness, CIMT) is associated with cardiovascular disease and stroke. Previous studies indicate that carotid intima-medial thickness is a significantly heritable phenotype, but the responsible genes are largely unknown. Hexose-6 phosphate dehydrogenase (H6PDH) is a microsomal enzyme whose activity regulates corticosteroid metabolism in the liver and adipose tissue; variability in measures of corticosteroid metabolism within the normal range have been associated with risk factors for cardiovascular disease. We performed a genetic association study in 854 members of 224 families to assess the relationship between polymorphisms in the gene coding for hexose-6 phosphate dehydrogenase (H6PD) and carotid intima-medial thickness.

**Methods:**

Families were ascertained via a hypertensive proband. CIMT was measured using B-mode ultrasound. Single nucleotide polymorphisms (SNPs) tagging common variation in the H6PD gene were genotyped. Association was assessed following adjustment for significant covariates including “classical” cardiovascular risk factors. Functional studies to determine the effect of particular SNPs on H6PDH were performed.

**Results:**

There was evidence of association between the single nucleotide polymorphism rs17368528 in exon five of the H6PD gene, which encodes an amino-acid change from proline to leucine in the H6PDH protein, and mean carotid intima-medial thickness (p = 0.00065). Genotype was associated with a 5% (or 0.04 mm) higher mean carotid intima-medial thickness measurement per allele, and determined 2% of the population variability in the phenotype.

**Conclusions:**

Our results suggest a novel role for the H6PD gene in atherosclerosis susceptibility.

## Introduction

Carotid artery intima-media wall thickness (CIMT), measured by ultrasonography, is a subclinical marker of systemic atherosclerosis. There is a direct relationship between CIMT and the risk of cardiovascular disease and stroke [1]. This association appears to be independent of “traditional” risk factors such as hypertension and diabetes. Previous studies indicate that CIMT is a significantly heritable phenotype [2].

Hexose-6 phosphate dehydrogenase (H6PDH) is a microsomal enzyme which is a component of the pentose phosphate pathway. It is thought to function in regenerating NADPH within the endoplasmic reticulum for use in steroid hormone and drug metabolism. H6PDH uses as substrate glucose-6 phosphate and the cofactor NADP(+), producing 6-phosphogluconate and NADPH. Although all molecular interactions of H6PDH are not as yet known, its role in supplying NADPH to, and so regulating the oxo-reductase activity of, 11-beta hydroxysteroid dehydrogenase type I (11β-HSD1), is well documented. NADPH generated by H6PDH is an essential cofactor for the action of 11β-HSD1 in reducing cortisone to cortisol, which takes place chiefly in the liver and adipose tissue [3]. 11β-HSD1 oxo-reductase activity has been implicated in the pathogenesis of several conditions related to atherosclerosis, including diabetes and the metabolic syndrome [4]. 11β-HSD1 inhibitors are an active area of pharmacological research in view of their potential as anti-diabetic or anti-obesity agents [5]. In view of its regulatory role on 11β-HSD1 activity, the H6PD gene, which encodes the H6PDH protein, is a plausible candidate gene for atherosclerosis susceptibility. No study to date, however, has examined whether genetic variation in H6PD is associated with CIMT. We investigated this question in a large family-based association study.

## Methods

### Population collection and phenotyping

Between 1993 and 1997, British Caucasian families were ascertained from hypertensive probands for a quantitative genetic study of cardiovascular risk factors. The local institutional review committee approved the study, and all subjects gave written informed consent. Two hundred and forty eight families with 1425 members were collected. The ascertainment strategy has been described previously [6] [7] [8], and further details are presented in Supporting Information S1. Between 1999 and 2001, families were invited to attend for further phenotyping. 955 family members (449 men and 506 women) out of a total of 1425 individuals who were invited attended. At this visit, carotid artery ultrasonography was performed by two sonographers using a 7.5-MHz linear array transducer (HP Sonos 5500). All measurements were made by one trained physician (BMM). The scanning protocol involved studying the right and left common carotid arteries, measuring far-wall IMT according to a standard method described by others [9] [10]. All scans were recorded on an optical disc for later off-line analysis. End-diastolic frames (one from each side of the body) were analysed for mean and maximal IMT, and the average reading from these two frames was calculated. Scans of sufficient quality were analysed with a previously described computerised edge-detection system typically delivering interobserver CVs of 2.5% [9]. As previously reported, intra-reader, inter-reader and inter-sonographer reproducibility was assessed, and found to be comparable to that achieved by other groups [2].

### Ethics Statement

The project was approved by the Central Oxford, and by the Newcastle and North Tyneside Research Ethics Committees, and all subjects gave written informed consent.

### TagSNP selection and genotyping

**Figure 1:**
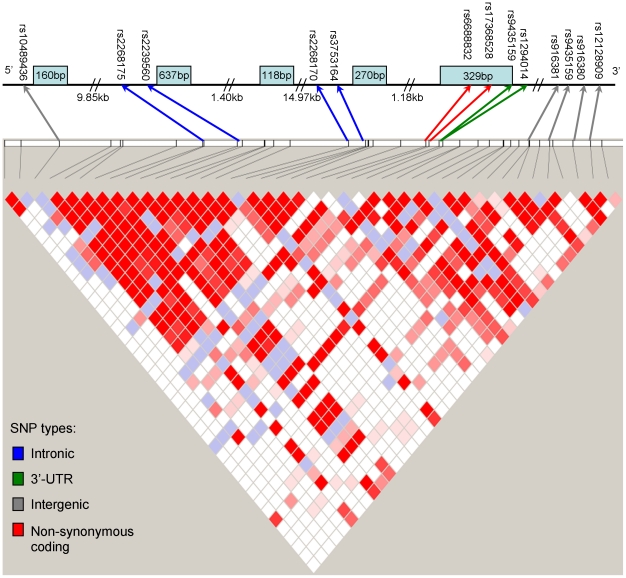
The H6PD gene, showing SNPs typed in this study. SNPs are shown in the context of Haploview output showing all SNPs typed in the CEU population of the HapMap project. Exons are represented as blue boxes. In the Haploview triangle plot D′ is represented, and stronger LD is indicated by red filled squares.

TagSNPs were selected from the phase 2 HapMap CEU data from the region of the H6PD gene and 15 Kb upstream and downstream (www.hapmap.org), using the Tagger utility of the Haploview program and these parameters: minor allele frequency >0.05; r^2^>0.8 [11]. 13 tagSNPs were required. A schematic view of the H6PD gene and the SNPs typed is presented in Figure 1. All TagSNPs were genotyped on a Sequenom MassArray MALDI-TOF platform using iPLEX Gold chemistry. Primer sequences and PCR conditions are presented in Supporting Information S1. Control individuals of known genotype were included in every plate, and 100 randomly selected samples were genotyped twice for each polymorphism. Genotyping was carried out blinded to phenotypic information. Mendelian inheritance of all the genotypes, and Hardy-Weinberg equilibrium for each marker, were checked using PEDSTATS [12]. Additional checks based on unlikely recombination patterns within families were carried out using the error-checking option in MERLIN [13]. Errors were corrected when possible by reference to the raw genotyping data, and when this was not possible genotypes were excluded from analysis.

### Statistical analysis

We examined the CIMT data for Normality; CIMT required log-transformation to adequately conform to a Normal distribution. We examined for the presence of significant covariates, and adjusted log-transformed CIMT for such covariates, using linear regression. Age, age^2^, sex, alcohol consumption (in units per week), smoking (graded current/former/never), exercise habit (graded none versus some regular exercise), diabetes, and hypertension affection status were considered as potential covariates of CIMT, and included in the model if they achieved significance at p<0.05. The log-transformed, covariate-adjusted residuals were then entered into the quantitative trait genetic association analyses, which were performed using a variance-components approach which takes account of shared polygenic effects in members of the same pedigree, implemented in the MERLIN package as previously described [13]. To make some allowance for the testing of multiple SNPs, we used the program QVALUE running on top of the statistical package R to determine q-values [14]. The q-value of a test measures the minimum false discovery rate that is incurred when calling that particular test significant; the approach taken by QVALUE involves using the vector of calculated p-values for all individual tests as input. We adopted an arbitrary FDR threshold of 0.05, that is, one in twenty of the associations passing this criterion are expected to be false. Since there was a degree of genotypic correlation due to LD between most of our tagSNPs (though a tagSNP strategy selects against strong inter-SNP correlation), this should be a conservative estimate.

### Bioinformatic evaluation of H6PDH P554L variant

The evolutionary conservation of the 554 residue between species was determined using Ensembl. The likely impact of the P554L non-synonymous SNP in the H6PDH gene on H6PD protein function was assessed using the PolyPhen prediction program.

### *In vitro* functional evaluation of H6PDH P554L variant

The *in vitro* effect of P554L on enzyme activity was assessed as described previously [15]. In brief, wild-type (WT) and mutant (P554L) H6PDH cDNA, contained in pcDNA3.1D/V5-His-TOPO (Invitrogen, Paisley, UK), were used to transfect a human embryonic kidney cell line (HEK 293), devoid of endogenous H6PDH. Three stably transfected cell lines were derived from three separate transfection experiments for WT and P554L, with cells mock transfected with empty vector used as a further control. Successful and equal transfection levels were confirmed using H6PDH-specific, quantitative PCR using commercially available assays (Target sequence NM_004285.3; Applied Biosystems Taqman Probe ID: Hs00188728_m1) used according to the manufacturer's instructions. H6PDH assays were performed on WT and mutant microsomes prepared from HEK 293 cells (ATCC, Manassas, USA) by spectrofluorometric detection of NADPH generation. Microsomes were permeabilized with 0.5% Triton X-100 and incubated in 50 mM glycine buffer (pH 9.0) at 37 C in the presence of 1 mM G6P and 0.4 mM NADP^+^. Absorbance readings were recorded using a luminescence spectrometer (excitation 340 nm, emission 456 nm), and readings converted to nmoles NADPH produced/min/mg of total microsomal protein.

## Results

### Demographics and phenotype distribution

There were 854 members of 224 families who had acceptable CIMT measurements. Demographics and phenotype distribution of these participants are shown in Table 1 (and the corresponding information for the entire population is presented in Supporting Information S1). As expected given the ascertainment on a hypertensive proband, participants had on average higher systolic and diastolic blood pressures than a general population. We used mean CIMT as the principal variable after preliminary analyses showed highly similar results for mean and maximal CIMT phenotypes, which were strongly correlated (r = 0.98). The distribution of the mean CIMT values lay mainly within the normal population range (median observed 0.76 mm, IQR 0.64–0.91 mm; normal range 0.4–1.0 mm). The distribution of the mean CIMT data was significantly skewed, and was therefore transformed by taking the natural logarithm, after which the distribution conformed to Normality. The significant covariates for log_e_ mean CIMT were age, age-squared, sex, alcohol consumption (in women only) and physical exercise. These covariates were all highly significant (p<0.005), and accounted for 38.9% of variability in log_e_ mean CIMT. Following adjustment for covariates, the heritability of log_e_ mean CIMT was 22% (p<0.001).

**Table 1:** Characteristics of the Population.

Variable	n	Minimum	Lower Quartile	*Median/Percentage	Upper Quartile	Maximum	†R^2^
Age (years)	854	18.7	37.7	51.0	59.9	88.1	-
Gender(female)	854			52.1			-
Hypertensive	802			41.5			-
Diabetes	438			2.1			-
Smoker	852			19.2			-
Take No Exercise	846			40.0			-
Alcohol Consumption (units per week)	852	0	0	3.5	12.0	80.0	-
Clinic Systolic Blood Pressure (mmHg)	738	86.0	122.3	135.0	154.0	226	28.8
Clinic Diastolic Blood Pressure (mmHg)	737	47.0	74.0	83.0	93.0	135.7	16.7
BMI (kg/m^2^)	846	17.2	23.3	25.5	28.3	51.8	14.9
WHR	835	0.63	0.78	0.85	0.91	1.20	49.6
Total Cholesterol (mMol/l)	777	2.6	4.8	5.5	6.3	10.6	18.0
IMT mean (mm)	854	0.42	0.65	0.76	0.91	2.17	38.9

† Proportion of variability explained by correction for covariates. All variables were log-transformed before correction, except WHR, to achieve approximate Normality.

*Medians are given for continuous variables and percentages for binary variables.

### Analysis of association between H6PD SNPs and CIMT

**Figure 2:**
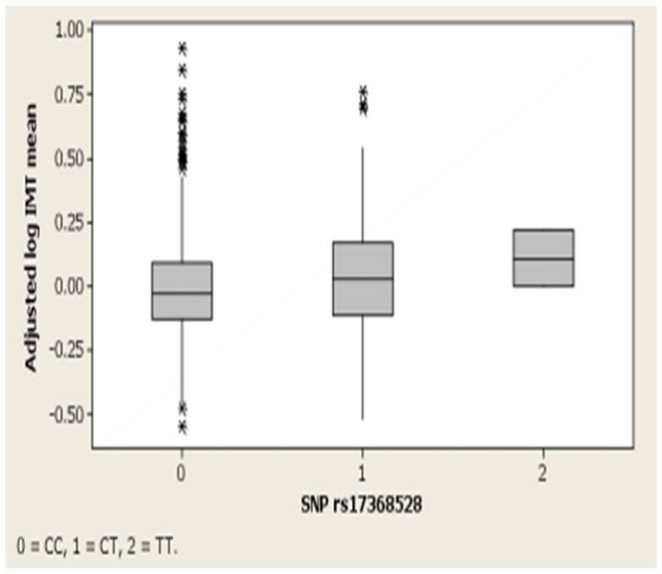
Boxplot of mean CIMT by rs17368528 genotype.

Genotyping was successful in over 97% of samples for all SNPs. There was no significant deviation from Hardy-Weinberg proportions for any SNP (all p>0.05). Minor allele frequencies of the typed SNPs varied from 0.09 to 0.49, and were in close correspondence with the HapMap data (CEU population) for all SNPs. As expected given the tagSNP selection strategy, correlation (r^2^) between SNPs was generally modest (Supporting Information S1). The estimated genotyping error rate was less than 1%. The rs17368528 SNP in exon 5 of H6PD was significantly associated with adjusted log [mean CIMT] (p-value for linear trend = 0.00065; Table 2), with carriers of the minor T allele having higher mean CIMT. However, the number of homozygotes for the T allele was small. Therefore, to rule out a disproportionate influence from rare outliers on the result a test comparing T allele carriers with non-carriers was also performed (that is, a dominant model); this was similarly significant (p = 0.007, Table 2). Genotype explained 2% of the variability in adjusted log [mean CIMT], or around 9% of the genetic variability in the phenotype. Simple exponentiation indicates each T allele increases mean CIMT by approximately 5%, which in our population would translate to around 0.04 mm. As expected, some of the other tagSNPs that were in LD with rs17368528 had weaker evidence of association with CIMT, but only rs17368528 remained significant at an FDR threshold of 0.05 using QVALUE. A boxplot showing the relationship between CIMT and rs17368528 genotype is presented in Figure 2.

**Table 2:** Association of CIMT with rs17368528 genotype.

	Mean (standard error, n) for each genotype at rs17368528			p-values for linear trend, and for CT+TT v CC
Variable	CC	CT	TT	
Adjusted log IMT mean	−0.0101(0.008, 616)	0.0402(0.018, 149)	0.1080(0.145, 2)	0.00065, 0.007

As the cohort was selected for blood pressure, it was particularly important to rule out any confounding effect of blood pressure on our results. While this would largely have been accomplished by our principal approach, which involved adjusting CIMT for any effect of blood pressure, we also carried out subsidiary analyses to test whether there was any effect of H6PD genotypes on blood pressure. No significant association of genotype with blood pressure, considered either quantitatively or qualitatively, was observed at any SNP typed.

### Bioinformatic analysis of P554L variant

**Figure 3:**
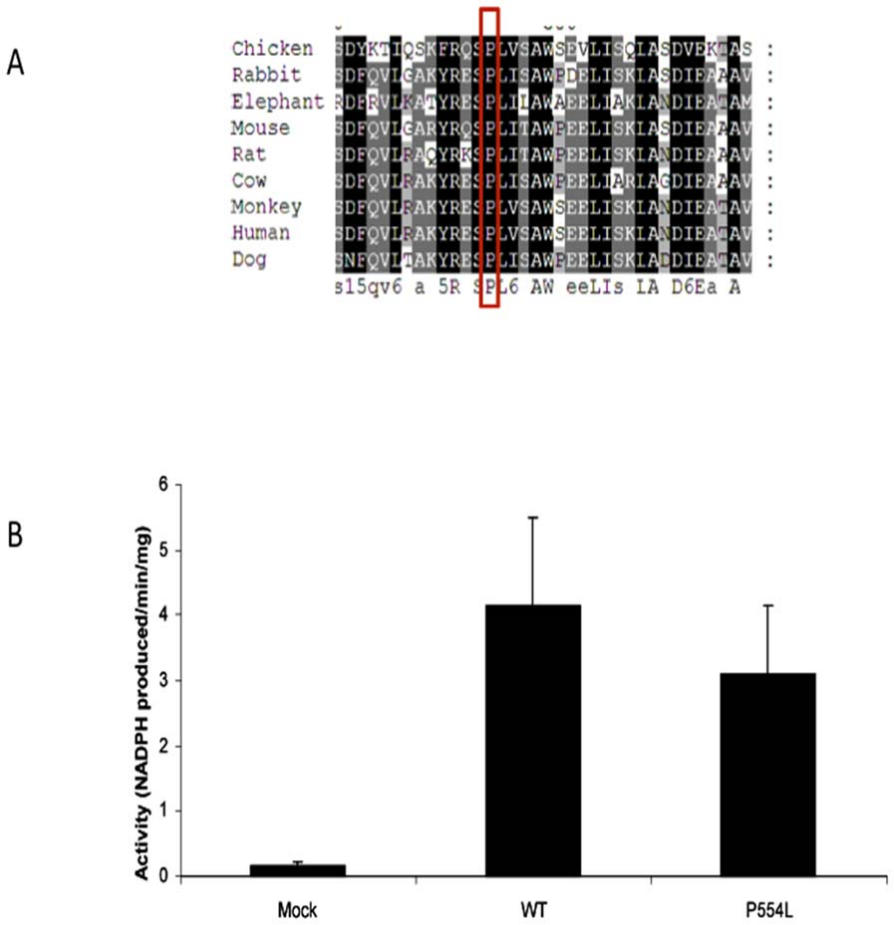
Bioinformatic and functional investigation of rs17368528. A) H6PDH protein sequence alignments indicating the strict conservation of Proline 554 (red box) between species. B) H6PDH activity assays performed on microsomal preparations from HEK-293 cells transfected with empty vector (Mock), and WT and P554L expression constructs. There was no significant difference in activity between WT and P554L.

The rare allele of the rs17368528 SNP results in a substitution of leucine for proline at position 554 of the H6PD protein (Pro554Leu). Bioinformatic analysis of this substitution using the prediction tool PolyPhen indicated it to be “probably damaging”. In addition, alignments of known H6PDH protein sequences revealed proline 554 to be highly conserved and therefore likely to be of functional importance (Figure 3A).

### *In vitro* functional analysis of P554L variant

The standard H6PD activity assay we performed did not detect any significant differences in enzyme function between WT and P554L variants with respect to their capacity to generate NADPH (Figure 3B).

## Discussion

We describe genetic association between a nonsynonymous SNP in exon 5 of H6PD and carotid intima-medial thickness. The association accounts for just under 2% of the total population variability, which represents just under 10% of the heritability of the phenotype in our cohort. CIMT was 5% higher per copy of the rare T allele at rs17368528. The rs17368528 SNP encodes a proline to leucine substitution in exon 5 of H6PD and is predicted to have a deleterious effect on protein function.

Although there have been many previous candidate gene association studies of CIMT (for example, over 100 are recorded in the Genetic Association Database; www.geneticassociationdb.nih.gov) none so far has, to our knowledge, investigated the H6PD gene. A systematic review of all published candidate gene studies of CIMT up to 2010 suggested modest association with the Apolipoprotein E epsilon2/epsilon3/epsilon4 isoform polymorphism only; this did not appear to be confounded by publication bias in favour of small positive studies. By contrast, review of the published literature for other commonly studied polymorphisms including the insertion/deletion polymorphism of the angiotensin-1 converting enzyme gene (ACE I/D), and the C677T SNP in the 5,10-methylenetetrahydrofolate reductase gene (MTHFR), showed evidence of publication bias likely to be of equal or greater magnitude than any true genetic effect [16].

Published data from association studies typing many thousands of SNPs that have included CIMT as a phenotype at present remain at low resolution and of comparable size to the present study. The Framingham Heart Study investigators obtained genotypes at 100,000 SNPs genomewide on around 970 individuals and observed evidence of association that approached genomewide significance levels at rs1376877 on chromosome 2q and rs4814615 on chromosome 20p. No significant association was reported in the region of H6PD, but rs17368528 was not represented on the chip used in that study [17]. Recently Lanktree and colleagues presented a multi-ethnic genetic association study of CIMT in a total of 898 people using a targeted, collaboratively designed, gene-centric 50,000 SNP chip covering some 2100 candidate genes for atherosclerotic cardiovascular disease, which demonstrated significant association with SNPs in the histone deacetylase 4 (HDAC4) and natriuretic peptide receptor A (NPR1) genes [18]. Many other genetic epidemiological cohort studies, including some with CIMT data, are presently genotyping this same chip and may replicate those findings, however, the chip does not include SNPs in H6PD [19]. Replication of our result by others will therefore await the result of genotyping large cohorts in which CIMT has been measured using denser SNP panels or of focused genotyping in the H6PD region.

We calculated that the increase in CIMT associated with homozygosity for the T allele at rs17368528 compared with homozygosity for the C allele is around 0.1 mm. Taken together, previous prospective studies suggest that differences of this order in IMT measured at baseline would be associated with an increase in relative risk for myocardial infarction and stroke over time of only around 1.10 [10] [20]. Association between H6PD genotypes and clinical endpoints of stroke or myocardial infarction have not been observed in large-scale genome-wide association studies of those endpoints conducted so far. However, studies published to date do not have adequate power to rule out effects on endpoints of the size that would be anticipated given the association we describe with the quantitative phenotype. Given the size of the effect we have detected, it seems unlikely that rs17368528 genotype would be useful in individualized risk stratification; however, our finding does point to a hitherto unrecognized potential role of H6PDH in atherosclerosis susceptibility.

Inhibition of H6PDH activity, and thus depletion of the ER luminal store of NADPH, sensitizes cells to oxidative stress and thus promotes apoptosis, which is a key process in the vessel wall in the initiation and progression of atherosclerosis [21]. Although PolyPhen analysis and sequence alignments suggested the Pro554Leu variant to be “probably damaging”, *in vitro* functional assays did not reveal any significant, large scale effects of this mutation on NADPH generation compared to WT H6PDH. There are however, certain caveats to the functional assay used in this study. Firstly, the method would not be able to detect subtle differences (decreases or increases) in the kinetic parameters that might arise due to this mutation, since the assay uses only crude cell extracts and measures only initial rates, with saturating substrate concentrations. Secondly, H6PDH is a bifunctional enzyme with both glucose-6-phosphate dehydrogenase activity (which is responsible for the NADPH generation) and 6-phosphogluconolactonase activity [22]. The P554L mutation is situated at the junction between the enzyme domains responsible for these two functions. The assay carried out in this study only directly measured NADPH generation (glucose-6-phosphate dehydrogenase activity) and not 6-phosphogluconolactonase activity, which is much more difficult to assay. 6-phosphogluconolactonase activity accelerates the hydrolysis of the reactive/toxic intermediate 6-phosphogluconolactone [23]. Any interference with the functionality of the second domain could also affect the generation of NADPH by the first domain. In order to carry out further analyses of the effect of the mutation on 6-phosphogluconolactonase activity, further experiments using highly purified protein will be required. Finally, H6PDH and 11β-HSD1 have been reported to have a direct protein-protein interaction which facilitates the reductive activity of 11β-HSD1 [24] and this could not be assessed in our assay. The precise residues involved in the interaction between the two proteins have not been fully elucidated, but it is possible that the P554L mutation may affect this interface ^23^. Further experiments will therefore be required to identify the specific mechanism whereby H6PD Pro554Leu affects atherosclerosis susceptibility.

Our result was obtained in a large study of families closely characterized for many potential confounders, for which it was possible to carry out adjustment before the genetic analyses. It is robust to a correction for multiple comparisons in which we adopted an FDR of 1 in 20. However, as with any hypothesis-originating study, replication in an independently ascertained cohort will be important in due course. Given the modest absolute size of the effect (0.1 mm difference between genotypes) and the technical demands of accurately measuring CIMT on large numbers of participants, this might be challenging. Pharmacological inhibitors of 11β-HSD1 are presently the focus of a great deal of interest because of their possible role in decreasing the risk of obesity and the metabolic syndrome. Our result suggests that more detailed investigations of the role of the endoplasmic reticulum redox system in atherosclerosis is warranted to fully understand the potential implications of such agents and design potential new compounds.

### Conclusions: Study Relevance

Pre-receptor regulation of glucocorticoid signaling is implicated in the development of obesity and the metabolic syndrome. Drugs to modulate the activity of this system are presently in clinical evaluation. Our result suggests that in addition to effects on the metabolic syndrome, this pathway has direct effects on the atherosclerotic process that may be of therapeutic importance.

## Supporting Information

Supporting Information S1 Supporting Information S1 contains Supplementary Methods, Supplementary Tables 1, 2, and 3, and Supplementary Figure 1.
(DOC)
