# Supplementary material for: Genotype at the P554L Variant of the Hexose-6 Phosphate Dehydrogenase Gene Is Associated with Carotid Intima-Medial Thickness
Source: PLoS One. 2011 Aug 12;6(8):e23248. doi: 10.1371/journal.pone.0023248 (PMC3155541; doi:10.1371/journal.pone.0023248)

**Supporting Information for Rahman et al. “Genotype at the P554L variant of the hexose-6 phosphate dehydrogenase gene is associated with carotid intima-medial thickness.”**

**Contents:**

Supplementary Methods: Additional details of population collection strategy

Supplementary Table 1: Sequenom primers and PCR conditions for genotyping H6PD SNPs

Supplementary Table 2: Characteristics of the study population

Supplementary Table 3: Marker characteristics in the entire population

Supplementary Figure 1: Linkage disequilibrium between typed markers

**Supplementary Methods**

Population Collection

Families were selected through a proband with essential hypertension whose systolic and diastolic BP were in the top 5-10% of the population distribution (defined as daytime ambulatory BP >140/90mmHg; three clinic BP measurements >160/95mmHg; or treatment with at least two antihypertensive medications). Secondary hypertension was excluded using the screening protocol applied in the hypertension clinic. Families were required to consist of at least three siblings (including the proband) clinically assessable for BP if DNA from a parent of the sibship was available, or at least four siblings if no parental DNA was available. Qualifying sibships could be in the generation of the proband, or the offspring. There was no requirement for additional members of the family to be hypertensive, but where additional members of the sibship were found to have hypertension (using the same criteria), families were extended and the spouses and offspring of hypertensive members also collected. The majority (64%) of the individuals in the family collection therefore have BP within the conventionally accepted “normal range”, and the family collection includes some extended families, though most are nuclear families. The median family size was 5 people, 60% of families comprising between 4 and 6 genotyped and phenotyped members. 71% of families were 2-generation and 29% were 3-generation. 84% of families had an assessable sibship in the generation of the proband, while 16% of families consisted of a proband and their nuclear family (spouse and children over 18 years) only. A full clinical history was taken, anthropometric measurements including height, weight, waist and hip measurement were made, 24-hour ambulatory blood pressure was measured according to a previously described protocol, (Dudley C, Keavney B, Casadei B, Conway J, Bird R, Ratcliffe P. Prediction of patient responses to antihypertensive drugs using genetic polymorphisms: Investigation of renin-angiotensin system genes. J Hypertens. 1996;14:259-262) and blood was drawn into a variety of anticoagulants for plasma and DNA analysis.

Supplementary Table 1: Sequenom primers and PCR conditions used for typing SNPs in H6PD gene

| **SNP** | **Location (bp)** | **Forward primer (5’→3’)** | **Reverse primer (5’→3’)** | **Annealing temp. /**  **Conc. of MgCl_2_** | **Size of PCR amplicons (bp)** |
| --- | --- | --- | --- | --- | --- |
| rs10489436 | 9208308 | ACGTTGGATGAATGAAGAGGGAAGCTCCTG | ACGTTGGATGTTTATCTGCCAGCCAAGAGC | 56.0ºC / 2.5mM | 114 |
| rs2268175 | 9223195 | ACGTTGGATGTATCTCACCCTCCCATTTCC | ACGTTGGATGCCTGCCTCTAAACAACCACA | 56.0ºC / 2.5mM | 110 |
| rs2239560 | 9227318 | ACGTTGGATGTTTGCCGCTTTCCTATGCTC | ACGTTGGATGTCTGCCTGGGAAAGTTAGAG | 56.0ºC / 2.5mM | 94 |
| rs2268170 | 9238434 | ACGTTGGATGCAGAGATTCCAATGCCTACC | ACGTTGGATGCTTAACTGTACCTCTGAGGG | 56.0ºC / 2.5mM | 110 |
| rs3753164 | 9240523 | ACGTTGGATGTCTGTAGGTGAGGGTGTGAG | ACGTTGGATGTATACAGGACAGTCCCAGAG | 56.0ºC / 2.5mM | 113 |
| rs6688832 | 9246497 | ACGTTGGATGGATATGGGATAAGAGGACGG | ACGTTGGATGTGTCCGATTACTACGCCTAC | 56.0ºC / 2.5mM | 88 |
| rs17368528 | 9246800 | ACGTTGGATGAGTGACTTCCAGGTCCTCAG | ACGTTGGATGAGCTTAGAGATCAGCTCCTC | 56.0ºC / 2.5mM | 100 |
| rs9435159 | 9247852 | ACGTTGGATGTATCAACCAGCACAACACGG | ACGTTGGATGTTCCCTGATTAACCTCAGCC | 56.0ºC / 2.5mM | 107 |
| rs1294014 | 9248263 | ACGTTGGATGGAATGAGGGCTGATAACAGG | ACGTTGGATGATTTCTGGAACCCTTTCCAC | 56.0ºC / 2.5mM | 83 |
| rs916381 | 9256144 | ACGTTGGATGTGCTGCAGCGTCCAGCAGTG | ACGTTGGATGAGAAGCAGGGCAAGGTCTGA | 56.0ºC / 2.5mM | 119 |
| rs2871656 | 9259704 | ACGTTGGATGTCAGCAGCGTGAAAACAGAC | ACGTTGGATGTGGAGGCCACAACACCAGAC | 56.3ºC / 2.5mM | 97 |
| rs916380 | 9263625 | ACGTTGGATGGATAAAGTCCTCTCTGTGCG | ACGTTGGATGGTTTCACCACTGTGAGCTTG | 59.0ºC / 2.5mM | 108 |
| rs12128909 | 9264790 | ACGTTGGATGAGGCTGAGGACTGCAGGAAG | ACGTTGGATGTCTTTGCTGCTCTGCAC | 56.0ºC / 2.5mM | 119 |

Supplementary Table 1 (continued)

| **SNP** | **Unique extension primer** |
| --- | --- |
| rs10489436 | TAAGCTTAAGGCAAGAAAC |
| rs2268175 | CCTCTAAACAACCACATAACCTT |
| rs2239560 | CCCACTTGCTAGAGATGAA |
| rs2268170 | AAGCCTTACTCCATCCTTTT |
| rs3753164 | TCGCAGGAGAGTTCCAA |
| rs6688832 | GACGCCTACAGCCCTGTGC |
| rs17368528 | GGCTCCTCGGACCAGGCGGAGACCAGC |
| rs9435159 | TCACGTCTCCTCTTGT |
| rs1294014 | ACCCTTTCCACAGTCTA |
| rs916381 | TATGGAGAGCAAAGCCA |
| rs2871656 | CCACAACACCAGACCCCGAAATCCTC |
| rs916380 | TTGTGAGCTTGTTCCTC |
| rs12128909 | CCGAGCTGCAGAACAC |

Supplementary Table 2: Characteristics of the entire study population

| Variable | n | Minimum | Lower Quartile | ^*^Median/Percentage | Upper Quartile | Maximum | ^†^R^2^ |
| --- | --- | --- | --- | --- | --- | --- | --- |
| Age (years) | 1425 | 18.7 | 35.7 | 50.9 | 60.9 | 90.7 | - |
| Gender(female) | 1425 |  |  | 52.4 |  |  | - |
| Hypertensive | 1287 |  |  | 39.9 |  |  | - |
| Diabetes | 775 |  |  | 2.3 |  |  | - |
| Smoker | 1423 |  |  | 22.0 |  |  | - |
| Take No Exercise | 1413 |  |  | 43.1 |  |  | - |
| Alcohol Consumption (units per week) | 1420 | 0 | 0 | 3.0 | 12.0 | 80.0 | - |
| Clinic Systolic Blood Pressure (mmHg) | 1178 | 86.0 | 121.3 | 134.0 | 153.0 | 226 | 27.2 |
| Clinic Diastolic Blood Pressure (mmHg) | 1177 | 47.0 | 73.7 | 82.0 | 92.0 | 135.7 | 19.7 |
| BMI (kg/m^2^) | 1402 | 16.7 | 23.1 | 25.4 | 28.2 | 51.8 | 15.2 |
| WHR | 1358 | 0.56 | 0.78 | 0.85 | 0.91 | 1.22 | 48.7 |
| Total Cholesterol (mMol/l) | 1289 | 2.6 | 4.8 | 5.6 | 6.4 | 12.7 | 18.2 |
| IMT mean (mm) | 854 | 0.4225 | 0.65 | 0.76 | 0.91 | 2.17 | 38.9 |

^†^Proportion of variability explained by correction for covariates. All variables were log-transformed before correction, except WHR, to achieve approximate Normality. ^*^Medians are given for continuous variables and percentages for binary variables.

Supplementary Table 3: Marker characteristics in the typed population

| **SNP** | **Position** | **Alleles** | **Region** | **ObsHET** | **PredHET** | **HWpval** | **%Geno** | **MendErr** | **MAF** |
| --- | --- | --- | --- | --- | --- | --- | --- | --- | --- |
| rs10489436 | 9208308 | C:G | Intergenic | 0.502 | 0.497 | 0.8816 | 98.6 | 0 | 0.464 |
| rs2268175 | 9223195 | C:T | Intron 1 | 0.417 | 0.407 | 0.651 | 98.9 | 0 | 0.285 |
| rs2239560 | 9227318 | C:T | Intron 1 | 0.279 | 0.263 | 0.1821 | 99.3 | 0 | 0.156 |
| rs2268170 | 9238434 | A:G | Intron 3 | 0.507 | 0.488 | 0.3729 | 98.8 | 0 | 0.422 |
| rs3753164 | 9240523 | C:T | Intron 3 | 0.528 | 0.5 | 0.1864 | 99.1 | 0 | 0.49 |
| rs6688832 | 9246497 | A:G | Exon 5 | 0.519 | 0.485 | 0.0992 | 97.5 | 0 | 0.086 |
| rs17368528 | 9246800 | C:T | Exon 5 | 0.192 | 0.179 | 0.0763 | 99.4 | 0 | 0.099 |
| rs9435159 | 9247852 | G:A | 3’ UTR | 0.402 | 0.375 | 0.4041 | 99.2 | 0 | 0.25 |
| rs1294014 | 9248263 | G:A | 3’ UTR | 0.394 | 0.38 | 0.4204 | 99.3 | 0 | 0.255 |
| rs916381 | 9256144 | G:C | Intergenic | 0.246 | 0.253 | 0.5167 | 99.4 | 0 | 0.149 |
| rs2871656 | 9259704 | G:A | Intergenic | 0.395 | 0.414 | 0.2888 | 99.1 | 0 | 0.292 |
| rs916380 | 9263625 | T:C | Intergenic | 0.225 | 0.221 | 0.765 | 99.1 | 0 | 0.126 |
| rs12128909 | 9264790 | C:T | Intergenic | 0.311 | 0.32 | 0.5542 | 99.1 | 0 | 0.2 |

Supplementary Figure 1: Linkage disequilibrium between SNPs genotyped in H6PD gene. Left panel shows D’and right panel r^2


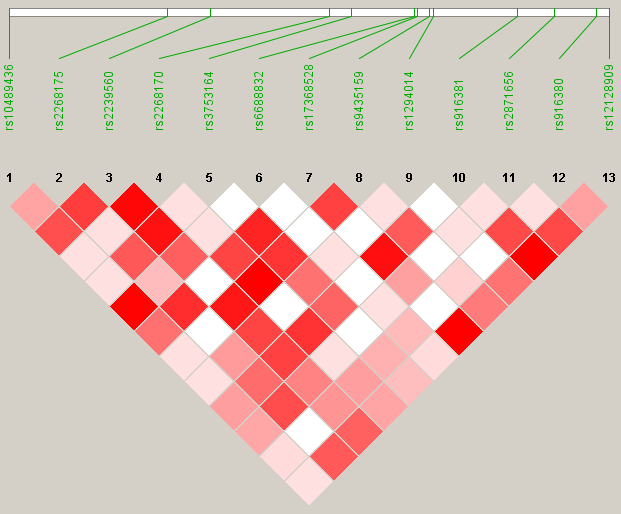

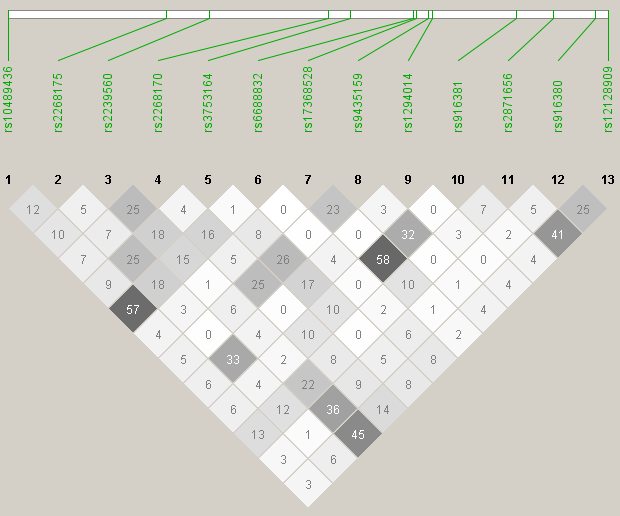

Supplement: Supporting Information S1 — contains Supplementary Methods, Supplementary Tables 1, 2, and 3, and Supplementary Figure 1. (DOC) [file pone.0023248.s001.doc]
